# Performance evaluation of the essential dimensions of the primary health care services in six localities of Bogota–Colombia: a cross-sectional study

**DOI:** 10.1186/1472-6963-13-315

**Published:** 2013-08-15

**Authors:** Paola A Mosquera, Jinneth Hernández, Román Vega, Jorge Martínez, Miguel San Sebastián

**Affiliations:** 1 Department of Public Health and Clinical Medicine, Epidemiology and Global Health. Umeå University, 901 87 Umeå, Sweden; 2 Postgraduate programs in Health Administration and Public Health, Pontificia Universidad Javeriana, Cr. 40 6-23 P.8 Bogota, Colombia

**Keywords:** Primary health care, Health services evaluation, Bogota

## Abstract

**Background:**

The high segmentation and fragmentation in the provision of services are some of the main problems of the Colombian health system. In 2004 the district government of Bogota decided to implement a Primary Health Care (PHC) strategy through the Home Health program. PHC was conceived as a model for transforming health care delivery within the network of the first-level public health care facilities. This study aims to evaluate the performance of the essential dimensions of the PHC strategy in six localities geographically distributed throughout Bogotá city.

**Methods:**

The rapid assessment tool to measure PHC performance, validated in Brazil, was applied. The perception of participants (users, professionals, health managers) in public health facilities where the Home Health program was implemented was compared with the perception of participants in private health facilities not implementing the program. A global performance index and specific indices for each primary care dimension were calculated. A multivariate logistic regression analysis was conducted to determine possible associations between the performance of the PHC dimensions and the self-perceived health status of users.

**Results:**

The global performance index was rated as good for all participants interviewed. In general, with the exception of professionals, the differences in most of the essential dimensions seemed to favor public health care facilities where the Home Health program was implemented. The weakest dimensions were the family focus and community orientation—rated as critical by users; the distribution of financial resources—rated as critical by health managers; and, accessibility—rated as intermediate by users.

**Conclusions:**

The overall findings suggest that the Home Health program could be improving the performance of the network of the first-level public health care facilities in some PHC essential dimensions, but significant efforts to achieve its objectives and raise its visibility in the community are required.

## Background

The Colombian General System of Social Security in Health (GSSSH) is based on an insurance market with different public-private provider combinations. Individuals are usually enrolled under one of two different regimes: the contributory regime, funded by payroll contributions, where formally employed and independent workers contribute a proportion of their incomes; and the subsidized regime, funded by general tax revenue, where poor people do not make any insurance contribution and are partially or fully covered depending on their poverty status [1,2]. Insurance companies from the contributory regime collect funds from the enrollees and outsource the provision of care through contracts mainly with private health care providers. Insurance companies from the subsidized regime receive funds from national transfers made by local health authorities and outsource the provision of care through contracts mostly with public health care providers.

Individuals in both, the contributory and subsidized regimes, choose their insurer and the health care providers from within the insurer’s network, and they receive a health benefits package. The contributory regime package covers all levels of care, while the subsidized covers primary care, as well as some inpatient and emergency care (40% less coverage than the contributory regime) [2].

The GSSSH offers a public health intervention package or Collective Intervention Plan (CIP—Plan de intervenciones colectivas in Spanish) which complements the mandatory health care insurance. Local health authorities provide health promotion and disease prevention services included in the CIP through contracts between Health Secretariats and public health providers [2].

Within this framework of health care segmentation [3-5] in 2004, the local government of Bogotá decided to apply a new initiative through the implementation of a Primary Health Care (PHC) strategy with a comprehensive approach. The comprehensive approach of PHC has been defined as the effective combination of promotive, preventive, curative and rehabilitative services. As an interactive model, primary health care encourages individuals and communities to be more involved in decisions about their health and its management [6]. Thus, PHC in Bogota was conceived as a strategic model for transforming health care delivery. The main purpose of the strategy was to guarantee the right to health and to achieve the highest possible level of population’s health with the emphasis on equity, solidarity and citizen self-reliance [7,8].

The essential elements of this strategy were: the introduction of a family and community orientation in the delivery of services; the reorganization and redistribution of primary care so that it became the gatekeeper of the health system; the implementation of an intersectoral response focused on solving community needs; and the promotion of social participation [7,8].

The core of the strategy, from the operational point of view, was the Home Health (*Salud a su Casa*) program. This program was implemented exclusively within the network of the first-level public health care facilities operating under the authority of the Bogota District Health Secretariat (DHS). According to the guideline, the program’s intervention began by prioritizing poor people classified as belonging to social strata^a^ 1 and 2, with the aim of gradual expansion to other strata. The program includes basic health care teams, comprised of a physician, a nurse, two community health workers, and an environmental technician who either provide intra- or extramural services. Twelve hundred families are assigned to each team in a geographically defined catchment area (micro-territories). These teams are supported by an expanded team consisting of a dental hygienist, dentist, physiotherapist, psychologist, and environmental engineer [8].

Financial resources for the implementation of the Home Health program are allocated from the DHS to public hospitals through the collective intervention plan [7]. Resources provided by the DHS, cover full-time salaries for the two community health workers and the environmental technician and part-time salaries for the nurse and the physician. The strategy assumes that hospitals will finance the rest of the professionals’ working time to guarantee a full health care team per micro-territory.

During the first year of the PHC implementation, the staff working at Home Health care teams and senior level officials involved in coordination and management activities in public hospitals, were trained to serve as multipliers through short courses and diploma courses offered by national and international universities [7]. Also the DHS in agreement with the National Apprenticeship Service (Servicio Nacional de Aprendizaje - SENA in Spanish) initiated a technical program to train community health workers and improve their skills in public health and health promotion strategies.

The program began its implementation in 2004 with the application of a household survey for the characterization of individuals, families and environmental health conditions in order to identify and to prioritize population needs and to design specific action plans according to the situation of the community. Once the needs have been identified, Home Health care teams provide health education at household level and when necessary, they refer people to social services and to their designated health care providers (contracted by the insurance company where they are enrolled). Priority cases (e.g. high risk pregnant women, disabled people) receive monitoring visits at home and are easier assigned to appointments in health care centers and hospitals. The program seeks to stimulate the demand for primary health care services and to facilitate access, intersectoral action and community participation through the intra- and extramural work of the teams [7].

By 2010, the program had achieved a 40.36% coverage (1,497,750 people) of the population in strata 1 and 2 in Bogotá, through the establishment of 358 basic health care teams. To respond to community needs, new health care facilities were created, opening hours of facilities were extended and an expansion of services was implemented [7].

While the above mentioned improvements are relevant, the main challenge for the Home Health program has been to provide health care according to the essential dimensions of primary care. These dimensions are first contact or gatekeeping, accessibility, longitudinality, comprehensiveness, coordination, family focus, and community orientation [9]. In this regard, one of the priorities for policy-makers (especially those interested in facilitating the expansion and development of the strategy) should be the analysis of the performance of the PHC strategy in relation to its essential dimensions, and its relationship to the improvement of the population’s health [10-12].

In Latin America, the performance of the essential dimensions of the PHC approach has been evaluated in Brazil. The evaluation of its Family Health program was done through the adaptation and validation of the Primary Care Assessment Tool (PCAT), where Brazilians established a useful and applicable methodology to different contexts within the developing world [10-12].

In Colombia, several studies have been published regarding the PHC strategy; however most of them aim to describe the historical process of implementation [13], the operational and management model of PHC, and the analysis of health outcomes and equity [14-16]. Regarding performance evaluation, a pilot study adapting the methodology validated in Brazil, has been reported on one locality in Bogotá. This study found a low performance in the dimensions of family focus and community orientation, and an intermediate performance in the coordination and comprehensiveness dimensions. The research also showed a positive association between the perceived health status and the performance of the essential dimensions [17].

This study was carried out in close collaboration with DHS and responds to its request to evaluate the performance of the essential dimensions of the PHC strategy implemented in six localities geographically distributed throughout Bogotá city. Additional secondary objectives were to compare the performance of the PHC dimensions between public and private healthcare facilities and to identify possible associations between the global performance index of the PHC and the self-perceived health status of users. The findings of this study helped to identify dimensions in need of improvement and inform the District Health Secretariat about the challenges ahead.

## Methods

### Study design

This is a cross-sectional study comparing the perception of performance of the essential dimensions of the PHC services from the perspective of participants in public health facilities where the Home Health program was implemented, and participants in private health facilities without the program implementation.

To assess the performance, the rapid assessment method validated by Almeida, Macinko et al. in Brazil was applied [10-12]. This methodology and its instruments are applicable to the evaluation of the Home Health program in Bogota since this strategy is based on the same essential dimensions assessed in the Brazilian experience.

### Instruments

The method uses four types of instruments to obtain the perspectives of the following categories of participants at facility level: users (adults, adult caregivers accompanying children, elderly people, and people with disabilities), professionals, and health managers (managers and supervisors). The instruments were translated from Portuguese to Spanish and pre-tested in one Bogotá locality in a pilot study in 2008 [17]. Some items were adapted (such as the list of services offered in primary care facilities) and additional questions were added to some of the dimensions reflecting specific features of Bogotá’s program (for example, questions about geographical accessibility, copayments to use services, the extent in which copayments mean that there is an economic barrier to the utilization of services, and the use of the electronic family record “PHC online” software).

Each instrument (questionnaire) used in this study contained a core set of about 90 to 100 questions. The instruments for users included questions related to socio-demographic variables such as age, sex, socioeconomic status, education, health system affiliation, basic housing, relationship with the user (the latter only for adult caregivers accompanying children, elderly people, and people with disabilities), health status (including self-perceived health status, limitations on performing normal activities, presence of diseases in the last 30 days, and use of medical care services in the last six months), and the performance assessment of the essential dimensions of PHC. The instruments for professionals and health managers included personal and professional information such as educational background, job functions, and working time experience, as well as the assessment of the essential dimensions of PHC.

### Primary health care dimensions

Based on the Brazilian methodology [10-12] and Starfield’s proposals [9,18], the essential dimensions evaluated were as follows: *first contact* or *gatekeeping—* the extent to which primary care serves as the entry point to other levels of care (in non-emergency situations); *accessibility—*the ability to use primary care services without financial, organizational, geographical and/or structural barriers; *longitudinality—*continuity of attention (person-centered) over time with a stable provider of services; *comprehensiveness*— the extent to which all essential services needed to provide for the majority of the population’s health needs are offered at primary care facilities; *coordination*— the extent to which primary care facilitates patients’ care between levels and with other important social services and sectors; *family focus—* the extent to which primary care considers the patient within the wider context, which includes the family environment, and the encouragement of the participation and support of the family; *community orientation*—how well primary care responds to community needs, encourages community participation and involves the population in the design processes of interventions; and, *professional training*—the necessary training for the people involved in providing care in the PHC approach.

In addition to the essential dimensions mentioned above, the dimension of *financial resources distribution* to be assessed by health managers was included. Items in this new dimension were oriented towards the exploration of whether the allocation of financial resources within the health facilities took into account health needs and the socio-economic differences of the population served, and whether special plans were designed to solve population’s needs.

### Population and sampling methodology

The study was conducted in Bogotá city, Colombia’s capital, which is divided geographically into 20 localities and four networks of health services. For this research, six localities from three networks of health services were selected. These were chosen for their large and diverse population (approximately three million people - 43% of the total population of Bogotá, and 68% of the total population classified as strata 1 and 2 belong to these six localities), because of their role as early adopters of the Home Health program, and because of their receptivity and acceptance of the research proposal.

The study universe consisted of all users of primary health care services, professionals, and health managers working at health care facilities in the six localities included in this study. The primary sampling unit (PSU) was the health care facility which allowed easy access to the primary care users who would be able to evaluate the services delivered. This also allowed each user to be linked with the place (public or private) where they received care.

It is important to highlight that users are assigned to a public or private health care facility depending on the type of regime they are enrolled with what reflects their labor situation. Thus, users from strata 1 and 2 without employment contract would be enrolled into an insurance company on the subsidized regime which in turn, outsources the provision of services to public care facilities; meanwhile, users from strata 1 and 2 with an employment contract (temporal, fixed or independent) would be enrolled into an insurance company of the contributory regime which in turn, outsources the provision of services to private care facilities. Public and private health care facilities offer basically the same services to people from strata 1 and 2 (outpatient services, oral hygiene and dental care, laboratory sampling collection and drug delivery) except that the public ones offer as well the Home Health program. Since no one could be characterized as part of the Home Health program in private health care facilities, and given that nobody uses more than one type of primary health care facility, there was no possibility of including a dual user.

The sample frame of the study consisted of all public and private health care facilities located in the six localities and registered at the Health Secretariat. All 50 public health care facilities identified were included in the survey. Private facilities were geo-referenced to identify their proximity to the public health facilities and make the characteristics of the populations comparable. All (71) private facilities located in the same area of influence as the public facilities were invited to participate and 46 (65%) agreed to be involved in the study.

For the selection of a sample of users within health care facilities, a stratified probability procedure was used according to the place in which the user received the services. Since the objective was to make comparisons between participants in public health facilities where the Home Health program was implemented and participants at private health facilities without the program implementation, the sample size was calculated using the formula below to detect the mean difference δ between the two populations with a significance level and power given:

$$n=\frac{4{\left(Z_{\left(1-\alpha /2\right)}+Z_{\left(1-\beta \right)}\right)}^{2}}{{\left(\delta /\sigma \right)}^{2}}$$

Taking into account the experience of Brazil [10-12], the following values were taken as references: α = 0.05, β = 0.15, δ = 0.5, y σ = 0.9. To control for clustering by type of healthcare facility (public and private), we multiply this formula by the design effect of 1.5. A target sample size was set at 3,000 users, comprising 1,500 at public facilities and 1,500 at private facilities. A sample of professionals and health managers were selected at random from the employee records of each facility. The sample set of professionals and health managers were 98 and 50 respectively (49 professionals and 25 health managers in each type of facility). The final sample size comprised 3,030 users (1519 at public facilities and 1511 at private facilities), 175 professionals (86 at public and 89 at private facilities), and 75 health managers (40 at public and 35 at private facilities), which was greater than the sample size proposed.

The instruments were applied by a group of trained facilitators through direct interview. Users were randomly selected in the waiting room of each health facility from among those with appointments in July and August 2010. The selection was made during five consecutive days in each locality, excepting weekends and holidays.

Interviewers considered two inclusion criteria for the selection of users: 1) in the public health care facilities, people who had been characterized as part of the Home Health program; in the private health facilities, people not identified as part of the Home Health program; and, 2) in both groups, people who had received medical care, at least once, in the place where they were recruited for the study.

For the selection of professionals and health managers, the inclusion criterion was staff members who were physicians or nurses at the health care facilities concerned. In the case of the public health care facilities, an additional criterion was to have worked at least six months in the Home Health program.

### Data analysis

The participants interviewed responded to each question using a scale with values ranging from 0 (*never*) to 5 (*always*). These questions were grouped according to the corresponding primary care dimension (access, comprehensiveness, etc.). For each group of questions, a dimension index was created for each type of participant (user, professional and health managers) and each type of facility (public, private). The resulting indices were then combined to obtain the global performance index (total PHC score).

Dimension indices were created using categorical principal component analysis (CATPCA) and the global performance index was calculated as the combination of the dimension indices using principal components analysis (PCA). CATPCA allows the identification of a component (index score) obtained as the combination of categorical variables (items or questions in this case) that have nominal or ordinal structure; this is an appropriate procedure in situations where the relation between the variables is not necessarily linear. This kind of analysis finds common empirical patterns between question scores, sorting common features into “components” and assigning a value (factor score) to each dimension. The score shows how strong the connections between the individual questions and the dimensions are. PCA has the same approach and follows a similar procedure to that of CATPCA but on the assumption that the variables have a linear relationship, which is the case for the dimensions within the global index.

The calculations of the indices of each PHC dimension as well as the global performance index were done by using procedures that took into account the complex nature of the sample design (complex sample module of SPSS 21.0 software) and controlled for possible clustering when calculating the variances of averages.

Some advantages of using CATPCA and PCA are that these techniques can handle a large number of variables to capture complex concepts. Using these methods, individual variables can be assigned a specific scaling; something that factor analysis does not allow [19-21]. An additional advantage is that the indices result from a weighted sum of each dimension and not from an arithmetic average where all dimensions have an equal weight. This ensures that indices give a better representation of the perception of all individuals.

Scores obtained by CATPCA were transformed linearly giving values from 0 to 100 using a formula to standardize scores (score minimum/score maximum- score minimum X 100). The corresponding values for standardized scores allowed comparisons to be made between questions and indices, and types of exposure and participants. A frequency distribution analysis of the indices’ scores was performed to find three separate cut-off points. The bottom 10% of the sample values—corresponding to scores less than 40—was considered a critical performance. Scores rated between 10% and 50% of the sample—values greater than 40 but less than 70—were considered an intermediate performance, and scores greater than 70 were considered as a good performance.

Data analysis included a socio-demographic characterization of the population sampled according to the type of facility (public or private). A global performance index (GPI) and the indices for each primary care dimension by type of facility and participant (users, professionals, and health managers) were calculated; then comparisons of the results obtained by type of facility were carried out. In addition, a multivariate logistic regression analysis was conducted to determine the possible associations between the performance of the PHC dimensions and the self-perceived health status of users. The model included the self-perceived health status (good vs no good) of all users (attending public and private facilities) and other explanatory variables (age, sex, educational level and socio-economic status) but not the type of facility variable because of its high collinearity with the GPI. Robust standard errors were estimated to take into account clustering within the type of facilities.

### Ethical approval

This study was approved by the Ethics Committee of the department of postgraduate programs in Health Administration and Public Health at Javeriana University.

## Results

Over two thirds of users in both types of facilities were women. With regard to age, the largest group in public and private health facilities were adults between 26 and 45 years of age (39.7% and 39.8% respectively), followed by adults over 46 years in the public health centers (29.8%), and young people aged between 12 and 25 in private ones (26.5%). The proportion of people in strata 1 and 2 attended in public health centers was 98.8% while in the private ones was 84.7%. All participants had their basic household needs met (electricity, sewerage, indoor bathroom) and both groups of users reported similar patterns of possession of refrigerators, TVs and cars; however, users at private facilities reported higher possession of goods such as radio and land lines, while users at public facilities reported higher possession of mobile phones. Users at public facilities had slightly less education than the users of private facilities (43.1% compared to 45.3% had completed high school), and they also rated their self-reported health status slightly lower than the users to private facilities (8.7% compared to 7.5% rated this as bad or very bad). The differences between users at public and private facilities in terms of basic household supplies, level of education, and self-reported health status were not significant (p > 0.05).

Among the professionals, over 60% of the interviewees were physicians in both types of groups. In the case of health managers, those belonging to private health care settings showed more specialized training, although the differences were non-significant (p > 0.05).

### Perceptions on the essential dimensions and global performance

The global performance index was rated as good (> 70) for all groups of participants without statistically significant differences among them, except in the group of professionals where the GPI was significantly better in private than in public facilities (Table 1).

**Table 1:** Comparison of the PHC dimensions indices by type of actor and exposure in six localities of Bogotá, 2010

**Dimension**	**Type of clinic**	**Users**				**Professionals**				**Health managers**			
		**Mean**	**Standard error**	**IC**		**Mean**	**Standard error**	**IC**		**Mean**	**Standard error**	**IC**	
Accessibility	Public facilities	44.92	1.21	42.52	47.32	72.33	3.12	68.92	75.74	85.09	2.32	80.44	89.75
	Private facilities	61.99*	1.59	58.83	65.16	82.74*	0.74	81.27	84.21	92.97*	0.66	91.66	94.33
Gatekeeping	Public facilities	84.71*	0.90	82.92	86.50	88.37	2.66	84.13	92.61	94.42	2.29	89.82	99.01
	Private facilities	81.11	1.41	78.30	83.92	86.14	2.03	81.76	90.52	88.82	5.26	80.09	100.00
Longitudinality	Public facilities	85.59	0.69	84.21	86.97	84.49	1.18	82.74	86.24	80.78	2.38	76.00	85.57
	Private facilities	86.14	1.04	84.07	88.20	87.67*	1.04	85.83	89.51	85.19	0.58	84.29	86.62
Comprehensiveness	Public facilities	87.07	0.48	86.12	88.02	89.44*	0.93	87.88	91.00	94.54*	0.73	93.09	96.01
	Private facilities	85.87	0.98	83.91	87.82	80.7	3.06	77.76	83.64	78.70	5.49	67.04	89.10
Coordination	Public facilities	81.18	1.42	78.34	84.02	94.33	1.82	93.01	95.65	88.13	1.00	86.11	90.14
	Private facilities	81.22	0.87	80.66	81.78	96.72*	1.61	96.24	97.20	83.39	3.51	76.51	90.61
Family focus	Public facilities	39.36	1.01	37.35	41.37	81.94	1.49	79.44	84.44	79.35	3.49	72.35	86.35
	Private facilities	39.72	1.42	36.90	42.54	91.04*	0.46	89.30	92.78	83.86*	3.82	76.35	91.68
Community orientation	Public facilities	40.26*	1.05	38.16	42.35	86.43*	2.16	83.23	89.63	74.04*	5.09	63.83	84.26
	Private facilities	26.25	0.90	24.47	28.04	60.02	2.14	55.90	64.14	61.05	4.30	50.38	67.64
Professional training	Public facilities	86.61	0.55	85.50	87.71	68.45	2.03	63.73	72.17	92.34	2.03	88.26	96.41
	Private facilities	86.13	0.91	84.32	87.94	76.47*	1.69	73.07	79.87	92.27	3.78	83.35	98.53
Financial resources distribution^	Public facilities	-	-			-	-			29.67*	1.00	27.66	31.68
	Private facilities									23.43	3.51	19.87	26.99
**Total score**	Public facilities	**70.60**	0.61	69.38	71.82	**68.61**	1.96	64.24	72.98	**81.13**	3.03	71.35	83.52
	Private facilities	**70.52**	0.88	68.77	72.27	77.18*	0.89	74.12	80.24	**78.00**	2.46	73.08	82.92

*Difference between public and private statistically significant (p < 0.005).

^Only assessed in health managers.

**Figure 1:**
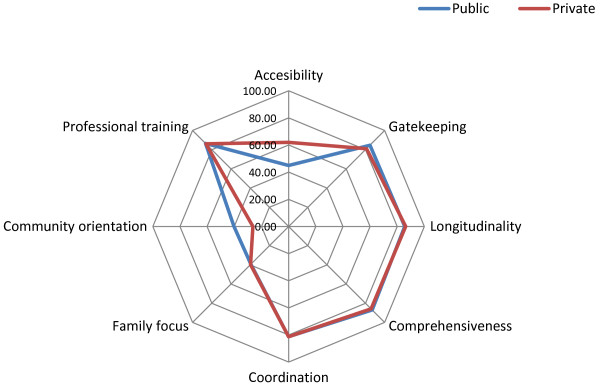
Performance evaluation of the PHC essential dimensions – Users.

Public and private users scored as critical the performance of *family focus*. *Community orientation* was rated as critical by users in private facilities and as intermediate by users in public facilities. Both public and private users graded a*ccessibility* as intermediate, and *gatekeeping, longitudinality, comprehensiveness, coordination* and *professional training* as good. *Gatekeeping* and *community orientation* were significantly higher among users in public facilities than among the private ones (Table 1 and Figure 1).

**Figure 2:**
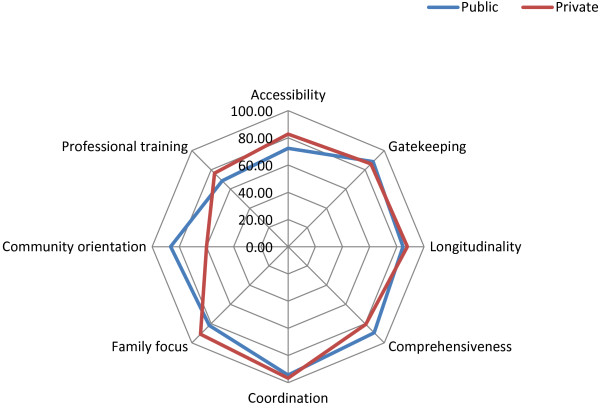
Performance evaluation of the PHC essential dimensions – Professionals.

Professionals rated almost all the essential dimensions as good performance, except *community orientation* in private facilities and *professional training* in public ones, which attained intermediate scores. *Comprehensiveness* and *community orientation* were significantly higher for public facilities than for private ones. *Accessibility, longitudinality, coordination, family focus*, and *professional training* were significantly higher for private facilities than for public ones (Table 1 and Figure 2).

**Figure 3:**
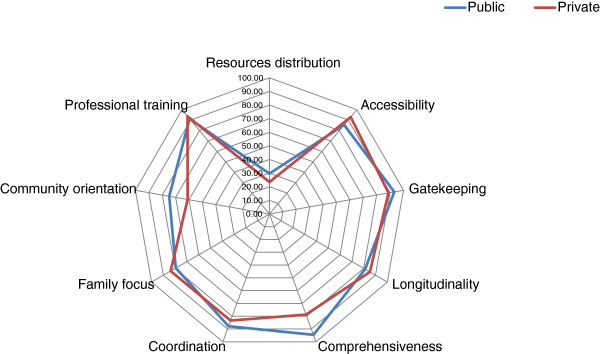
Performance evaluation of the PHC essential dimensions – Health Managers.

Health managers assigned a good performance to all essential dimensions except *community orientation* in private facilities (intermediate performance), and *financial resources distribution*, which was scored as critical by both public and private health managers. *Comprehensiveness, community orientation* and *financial resources distribution* were significantly higher for public facilities than for private ones while *accessibility* and *family focus* were significantly higher for private facilities (Table 1 and Figure 3).

Health managers and professionals’ scores were generally better than those of the users. The three groups coincided on significant differences in favor of the public health facilities in *community orientation*, and in favor of the private health facilities in *accessibility*. In general, with the exception of professionals, the differences in most of essential dimensions seemed to favor the public health care facilities where the Home Health program was implemented.

### Association between self-perceived health status and global performance index

The analysis showed that self-perceived health status was positively and significantly associated with the GPI of the PHC. Even after including other explanatory variables of perceived health status, such as education, economic index, gender and age, the association retained its significance (Table 2).

**Table 2:** Multivariate logistic regression analysis between users’ health perception and GPI adjusted for potential confounders in six localities of Bogotá 2010

	**Coefficient**	**Standard error**	**IC 95%**		**OR**	**IC 95%**	
Global Performance Index*	0,010	0,003	0,004	0,016	1,01	1,004	1,016
Sex	0,061	0,099	−0,133	0,255	1,06	0,875	1,291
Age*	−0,030	0,002	−0,034	.0,025	0,97	0,966	0,974
Educational level*	0,026	0,008	0,010	0,041	1,03	1,010	1,042
Economic Index	0,006	0,003	0,000	0,011	1,01	1,000	1,012

*Difference statistically significant (p < 0.005).

## Discussion

The first key finding of this study relates to the different perceptions of the PHC dimensions that professionals and health managers have from the users. In this regard, it is important to note the discrepancy between the perception of users and the other two groups of participants when it comes to *community orientation* and *family focus*, as these dimensions received a critical score from users and a good rating from professionals and health managers. A similarly discordant situation was observed in relation to *accessibility,* which was assigned an intermediate performance by users and a good grade by the other two groups.

This result highlights the importance of taking into account the perspectives of different participants in order to obtain a complementary view and improve the validity of the results. The analysis of the different perceptions in combination was helpful in that it enabled some critical areas to be identified which were not necessarily perceived by a specific group, and because it enabled the weakness of some dimensions to be highlighted.

Another important finding is that based on the users and health managers’ experience, no difference in the global performance between the two types of health care services was observed. Given that the Colombian public health care sector has been marginalized in the last years in terms of investments, public hospitals have been facing financial crises [22], and the private sector is perceived to perform better in some aspects [23,24]; we interpret this finding as a positive impact of the Health Home program in the services provision for the vulnerable populations of strata 1 and 2 at the first level of care. However, there is an ongoing discussion about this topic, and recent evidence suggests that private health providers might not be more efficient, accountable or medically effective [25]. Further monitoring of the performance will be needed in the future to contrast the findings of this study.

The results of this analysis carried out in six localities in Bogotá confirmed the preliminary results of the pilot assessment in one locality of the city [17], which found that the lowest rated dimensions were *family focus, community orientation, accessibility* and *financial resources distribution*. Similar results have also been reported by the studies conducted on the Brazilian Family Health program where a low performance score in *family focus, community orientation* and *accessibility* was reported [10,12,26,27].

The low performance of family *focus* and *community orientation* can partly be explained by the instruments used, as the questions asked are related to the relationships between users, professionals and health facilities, instead of the relationships between users and community health-workers, who are those who interact with the families and communities [27]. Other explanations could include the fragmentation and segmentation of the Colombian health system itself, where the structure allows different members of a family to belong to different regimes; thus, some key informants would not be aware of the family and community services [28]. In addition, professional and technical training on *family focus* and *community orientation* could be considered as weak because the educational and training programs are primarily focused on individual curative approach [14,17]. To this regard, some studies have pointed out the need to strengthen the training and re-training processes [29,30], and also overcome the problem of staff turnover (which means job instability), in order to improve *family focus* and *community orientation*[7,14,29].

The World Health Organization has also identified the promotion and strengthening of *family focus* and *community orientation* as a foundation for a comprehensive PHC strategy because of the close relationship of these dimensions with the possibilities for reinforcing intersectoral action and community participation [29,31]. *Family focus* and *community orientation* are also elements that, when working together in an articulated way, allow the PHC to act on the social determinants of health [7].

The low performance score assigned to the *financial resources distribution* could be due to the lack of a stable and clearly allocated source of funding for ensuring the comprehensiveness and sustainability of the strategy. Although the Collective Intervention Plan provides some funds to the Home Health program, financing is still a great challenge. Resources allocated to public health facilities are not enough to provide comprehensive care that reflects the individual and collective dimensions of population’s needs and for that reason, it has not been possible for health managers to move towards autonomy in the distribution of resources, taking into account the socio-economic differences of the population. Resolving this issue, although related to the health system structure itself, is an essential pre-requisite for the implementation of a successful comprehensive PHC strategy [28,31].

Regarding *accessibility*, the findings suggest that the efforts of the public health care network—through strategies such as extending opening hours including at weekends, making available mobile health care units, providing transportation and non-copayment for priority groups—are still insufficient, highlighting the great challenges in resolving geographic barriers and making organizational changes. The consequences of 20 years of privatization of the health care system in Colombia [32,33] might also have contributed to the low score of *accessibility*, in spite of the investments the Health Secretariat made to improve this dimension. Continuous efforts in this direction should be maintained.

The results of the association between the dimensions of performance and the self-perceived health status of the surveyed population are consistent with other studies [17,34] where the association, although discrete, is maintained even in the presence of factors known to be predictors of health status, such as socio-economic and contextual variables.

### Study limitations

The present study has certain limitations that need to be taken into account when considering its results. Regarding its design, the study included only the perceptions of those people that had used the health services, which could overestimate the ratings of some dimensions, especially *accessibility* and *gatekeeping*[35]. The cross-sectional design could not show whether the performance of the dimensions had improved over time and, if so, whether this has occurred in response to the implementation of the Home Health program. In addition, the multivariate analysis between the global performance index and users’ perception of health status could not establish causality and did not intend to suggest attribution to a provider’s action/inaction.

We are aware that the study results are not necessarily generalizable to different localities than those included in this analysis. The inclusion of 65% of the private health facilities could mean a lack of representation of the population in this group, and this might have influenced the dimensions’ scores. Moreover, the adaptation of the Brazilian methodology as well as the measurement of the scale of indices has not been validated and the psychometric properties of the instruments were not assessed, which reduces the reliability of the results.

Other limitations which might be considered include the partial comparability of the users, because those attending the public healthcare facilities had a lower socio-economic status than those attending the private ones. The use of CATPCA and PCA, where a weighting was given to the different items, might have resulted in overestimated or underestimated ratings. Also, the cut-off points to discriminate between a critical, intermediate and good performance may have caused a misclassification of some of the assessed dimensions.

## Conclusions

The overall findings suggest that the Home Health program could be helping to improve the performance of the first-level public health care facilities network in the essential dimensions of the PHC, but significant efforts are still required to achieve its objectives and visibility in the community. In line with the Brazilian experience, the methodology employed has been shown to be an easy, rapid and accessible tool for assessing and monitoring the performance of the primary health care strategy.

The ratings assigned to the dimensions by different participants confirm the need to strengthen family focus, community orientation, financial resources distribution, and access; these are key issues to address in achieving a comprehensive PHC aimed at responding adequately to the population’s health needs.

The above challenges call for transforming the fragmented and segmented health system, overcoming the financial logic of the Colombian health system, and returning to a territorial health care perspective best suited to the population’s needs. In addition to the aforementioned changes, this requires the implementation of health and social policies which focus on the social determinants of health, have well-trained and motivated human resources at their disposal, and have available a stable source of financial resources.

## Endnote

^a^Strata classify socioeconomic groups from 1 to 6, 1 being the lowest and 6 the highest. This classification determines the taxes and prices of utilities (gas, water, electricity) as well as access to health services, among others.

## Competing interests

The authors declare that they have no competing interests.

## Authors’ contributions

PM, JH, RV and JM conceived the study, participated in the data collection, analysis, interpretation of the data and drafted the manuscript. MSS contributed to the analysis and interpretation of the data and revised the manuscript for clarifications. All authors approved the final draft. All authors read and approved the final manuscript.

## Authors’ information

PM. Psychologist, specialist in epidemiology, Master in Social Policy and PhD student at the Department of Public Health and Clinical Medicine, Epidemiology and Global Health, Umeå University. 901 87 Umeå-Sweden. Research Associate at the programs in Health Administration and Public Health. Pontificia Universidad Javeriana. Cr. 40 6–23 P.8 Bogota-Colombia. Email: paolamosquera@gmail.com. JH. Medical Surgeon, Master Health Systems. Research Associate at the programs in Health Administration and Public Health. Pontificia Universidad Javeriana. Cr. 40 6–23 P.8 Bogota-Colombia.

Email: jinnether@hotmail.com. RV. Medical Surgeon, Master in Health Administration and Social Security and PhD in Health Administration. Professor at the postgraduate programs in Health Administration and Public Health. Pontificia Universidad Javeriana. Cr. 40 6–23 P.8 Bogota-Colombia. Email: rrvega.romero@gmail.com. JM. Mathematician, Master in Statistics, Master in Applied Mathematics, PhD in Statistics. Research Associate at the postgraduate programs in Health Administration and Public Health. Pontificia Universidad Javeriana. Cr. 40 6–23 P.8 Bogota-Colombia. Email: j.martinez.collantes@gmail.com. MSS. Associate professor in international health. Department of Public Health and Clinical Medicine. Epidemiology and Global Health. Umeå University. 901 87 Umeå-Sweden. Email: miguel.sansebastian@epiph.umu.se

## Pre-publication history

The pre-publication history for this paper can be accessed here:

http://www.biomedcentral.com/1472-6963/13/315/prepub
